# Precipitating factors and targeted therapies in combating the perils of sickle cell disease--- A special nutritional consideration

**DOI:** 10.1186/s12986-016-0109-7

**Published:** 2016-08-08

**Authors:** Shahida A. Khan, Ghazi Damanhouri, Ashraf Ali, Sarah A. Khan, Aziz Khan, Ahmed Bakillah, Samy Marouf, Ghazi Al Harbi, Saeed H. Halawani, Ahmad Makki

**Affiliations:** 1 King Fahd Medical Research Center, King Abdulaziz University, P.O. Box 80216, Jeddah, 21589 Kingdom of Saudi Arabia; 2 National Brain Research Center, Manesar, Gurgaon, 122051 India; 3 Department of Medicine, SUNY Downstate Medical Center, 450 Clarkson Ave., Brooklyn, New York 11203 United State of America (USA); 4 Department of Hematology, King Fahd Hospital of the Armed forces, Jeddah, Kingdom of Saudi Arabia; 5 Department of Hematology, Soliman Fakeeh Hospital Jeddah, Jeddah, Kingdom of Saudi Arabia; 6 Department of Hematology, Umm Al Qura University, Faculty of Medicine, Makkah, Kingdom of Saudi Arabia; 7 Department of Medical Laboratory, King Fahd Hospital of the Armed forces, Jeddah, Kingdom of Saudi Arabia

**Keywords:** Sickle cell disease, Hydroxyurea, Vaso-occlusive crisis, Nutrient deficiencies, Nutritional approaches

## Abstract

Nutritional research in sickle cell disease has been the focus in recent times owing to not only specific nutritional deficiencies, but also the improvements associated with less painful episodes. Though hydroxyurea remains the drug of choice, certain adverse health effects on long term supplementation makes room for researches of different compounds. Macro and micro nutrient deficiencies, along with vitamins, play an important role in not only meeting the calorific needs, but also reducing clinical complications and growth abnormalities. Symptoms of hyper protein metabolism, increased cell turnover, increased cardiac output, and appetite suppression due to enhanced cytokine production, might give us leads for better understanding of the mechanisms involved. Different nutritional approaches comprising of traditional herbal therapies, antioxidants, flavonoids, vitamins, minerals etc., reducing oxidative stress and blood aggregation, have been tried out to increase the health potential. Nutritional therapies may also serve complementary to the newer therapies using ozone, hematopoietic stem cell transplantation, antifungal medications, erythropoietin etc. Herein we try to present a holistic picture of the different patho-physiological mechanisms, and nutritional strategies adopted.

## Background

Sickle cell disease (SCD) one of the commonest prevalent autosomal recessive disease around the globe [1], is an inherited hematological disorder wherein, the oxygen-carrying molecule namely hemoglobin (Hb) present in the red blood cell, is defective. A mutation in the in the 6^th^ codon of the 11^th^ chromosome of the β globin chain, renders the amino acid valine to be substituted by glutamic acid resulting in HbS, the sickle cell haemoglobin [2].

The deoxygenated HbS undergoes hydrophobic interactions to form rod-like structures which are clusters of hemoglobin protein stuck to each other. These long fibers push the cell membrane out of shape causing the whole cell to become rigid,take a sickle shape, become deformed and adhere to the endothelium of blood vessels producing vasospasms,vasoconstriction, and triggering inflammation [3, 4] Adhesion is also significantly affected by alterations in hydration of erythrocytes. This changes their cellular tone and the cells become sickle shaped [5]. These red blood cells (RBC’s), due to their increased viscosity, sludge in the circulatory system obstructing microvasculature [6], producing oxygen deficiency at the target tissue/ organ. This causes tissue damage leading to ischemia and infarction and a compromised reduced life span [7].

In most of the cases, it requires immediate hospitalization and medical intervention with anti-inflammatory drugs, non-steroidal analgesics, hydroxyurea (HU), opioid analgesics, rehydration and in severe cases transfusion [8] which may cause other long term side effects.

Though born with a normal weight, children affected by SCD show weight deficits by year one which continues until adulthood accompanied by delayed skeletal maturation in both sexes and a delayed menarche in girls [9].

Fetal hemoglobin (HbF) is found in patients with SCD at different levels and is known to reduce the severity of symptoms of the disease. Hydroxyurea therapy has gained momentum as it raises levels of HbF accompanied by decreased morbidity. Though hydroxyurea is a potent HbF inducer in adults and children, it does not possess the ideal combination of efficacy, safety and ease of use [10]). This calls for further researches for compounds to alleviate the pain and improve the condition of patients with SCD. Several approaches have been made to prevent this disease or reverse the sickling phenomenon either through technological applications or the usage of compounds which affect the Hb molecule directly which are enlisted in the given Table 1.

**Table 1:** Drug candidates exhibiting benefits in sickle cell disease

Drug name	Action mechanism	Reference
Butyrate	HDAC inhibition, mood stabilization	[110, 111]
Decitabine	DNA demethylation	[112]
Trichostatin A	Increases HbF level; decrease adhesion of cells to vessel wall	[113]
Pomalidomide	Gammaglobin activation by Histone deacetylase	[114]
Senicapoc	Improves RBC hydration	[115]
Nitric Oxide	Increase NO	[116]
Tinzaparin	Decreases P-selectin-mediated acute pain episodes	[117]
6R-BH4	Increases NO; improve endothelial function	[118, 119]
Sidenafil	Increases NO	[116]
Eptifibatide	Decreases platelet aggregation and decreases CD40 ligand release	[120]
Statins	Improves endothelial function	[121]
Dexmethasone	Decrease inflammation	[122]
Nix-0699	Uncertain, but inhibits acute painful crisis	[44, 123, 124]
Intravenous immunoglobulin (IVIG)	Decreases the number of leukocytes & acute pain episodes	[44, 123, 124]
Vorinostat, panobinostat	HDAC inhibition	[113]
GMI-1070	Pan-selectin inhibitor	[45]
Propranolol	Inhibits RBC adhesion to the endothelium	[125]
Regadenoson	A2AR agonist, blocks iNKT activation	[126]
Zileuton	5-lipoxygenase inhibitor, used in asthma	[127]
Fructose-1,6-diphosphate (FDP)	Reduces ischemia–induced tissue damage	[128]
Prasugrel	ADP receptor blockade	[129]
MP4CO	PEG carboxy-hemoglobin	[130]
Acetyl-L-carnitine	Decreases lipid peroxidation	[90]
Alpha-lipoic acid	Inhibits NFkB, increases glutathione	[131]
NAC	Increased glutathione	[132]
Omega-3 fatty acids	Decreases VOC events	[18]
Glutamine	Increases NADPH	[133]
IV magnesium	Vasodilatation	[134]
Aes-103	Binds sickle hemoglobin and shifts oxy-hemoglobin dissociation curve to the left	[90]
Poloxamer-188	Non-ionic surfactant, improves micro-vascular flow	[135]
L-arginine	Substrate for NO	[116]

*HbF* fetal hemoglobin, *NO* nitric oxide, *RBC* red blood cell, *IVIG* intravenous immunoglobulin, *HDAC* Histone deacetylases, *Nix-0699* Niprisan, *GMI-1070* Rivipansel, *FDP* Fructose-1,6-diphosphate, *NADPH* Nicotinamide adenine dinucleotide phosphate, *A2AR* Adenosine A_2A_ receptor, *PEG* Polyethylene glycol, *ADP* Adenosine diphosphate, *iNKT* Invariant natural killer T cells

Few drug candidates may exhibit multiple mechanisms of action

Supplementation of oral antibiotics for a definite period in childhood to prevent pneumococcal infections is generally practiced in SCD patients. Also it has been observed by the Cochrane Reviewers that prophylactic penicillin considerably reduces the risk of pneumococcal infections and it is related with negligible side effects in SCD homozygous children, [11, 12]. The broad-spectrum antifungal drug Clotrimazole appears to be a well-tolerated drug, with few adverse reactions due to drug resistance in SCD patients, who are immune-compromised. Its metallic complexes have been also shown to exhibit improved efficacy [13]. Pharmacological agents targeting signaling molecules are also being tried out [10]

Though morphine is the opioid of choice, [14] it has been linked to pediatric acute chest syndrome (ACS) in patients hospitalized with severe pain [15], when administered intravenously. The risks of newer therapies using blood and bone marrow stem cell transplant though curative, outweigh the benefits, thereby limiting its usage because they are expensive and unaffordable to the vast majority of affected patients [16].

Multiple nutrient deficiencies have been observed with increased severity of the disease. Prevention of complications is expected from ongoing research in the nutritional scenario. Nutritional intervention with potential key nutrients has been in focus in recent times owing to specific nutritional deficiencies [17] and improvements in SCD associated painful episodes. Nutritional interventions to correct the existing cell membrane structure, composition and function could provide additional benefit to the new preventing and curative aspects of SCD. For example fish oils containing eicosapentaenoic acid (EPA) and docosahexaenoic acid (DHA) have been found to prevent the blockage of blood flow which could help in alleviating the problems associated with SCD [18]. Reduction of the vaso-occlusive crisis using zinc and piracetam are also quite encouraging, but larger cohorts, and/or longer term multi centric trials over a period of time are required to evaluate their efficacy [19]. A prospective nutritional approach with potentially active molecules like antioxidants might also be of benefit [20].

Interventions that are evidence based, sustainable, affordable and well incorporated into the healthcare structure should therefore be looked into. This review discusses the pros and cons of this much sought after issue in today’s context.

### Nutrient insufficiencies/deficiencies

**Fig. 1:**
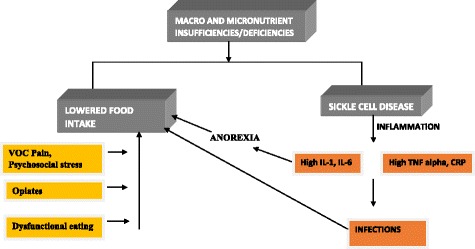
Nutrient deficiencies contributing to the vicious cycle in sickle cell disease

Nutritional intake has been found to be quite poor in SCD patients, and a serious need to correct the situation is implied [17]. Multiple nutrient insufficiencies or deficiencies of some micronutrients, vitamins, antioxidants and certain lipid constituents have been shown to be prevalent in patients with SCD and associated with increased disease severity of the disease (Fig. 1). Many complications associated with the disease, such as growth retardation, delayed sexual maturity, and a weak immune system, could be considered partly due to nutritional deficiencies [21]. In an important study Mandese et al. observed a significant relationship between body weight, body mass index (BMI) and either concentration of Hb or severity of the disease. They found that many of the nutritional components (macro and micro nutrients) are inadequate and affect the days of hospitalization for SCD patients. Also the concentration of HbF was negatively correlated with some of the nutrients like lipids, vitamins and minerals. They observed maximum nutrient deficiency in intake of Ca, Fe, vitamin B1 and C, while carbohydrates, lipids & vitamin B2 were moderately insufficient [22]. A study by Martyres et al. stressed out for the need of larger sample size to establish the relationship between nutrient deficiency and the severity of SCD [23].

Researchers have found that markers of elevated oxidative stress and inflammation are expressed in adolescents with SCD even in a steady state, which correlated with their resting energy expenditure (REE) [24]. Experimental studies show an imbalance in the chronic state of oxidant and antioxidant factors in red cells of SCD patients, resulting in an increase of oxidative stress and hemolysis [25].

### Role of macronutrient deficiencies

An increased plasma level of some acute inflammatory cytokines is observed in mice with sickle cell anemia (SCA) in response to infection. Supplementation of high protein diet resulted in decreased infection which can provide a clue to decreasing the incidents of infection in children with SCA. An increase in TNF -α, TNF- γ, IL-10, and IL-4 cytokines has been noted in the murine model of human SCA which is alleviated by supplementing a high protein diet [26]. In another study, decreased levels of CRP and IL-6 are observed on feeding a high protein diet [27].

Arginine plays an important role in SCD pathogenesis as insufficient / low global arginine bioavailability (GAB) is linked with many complications of the disease. Several mechanisms are proposed for arginine dysregulation in SCD patients. By inhibiting Gardo’s Channel activity arginine decreases the RBC density in transgenic SCD mice [28].

There is increased activity of arginase, increase in asymmetric dimethyl arginine, alteration in arginine transport mechanism and discrepancy in NO synthesis in these patients. Arginine supplementation through exogenous sources can restore low GAB. Arginine supplementation is quite successful in treating patients with leg ulcers, pulmonary hypertension and pain. Arginine supplementation along with hydroxyurea increases level of HbF and nitrite [29]. Preliminarily outcome of phase 2 clinical trial showed that reduced arginine induces pain and vaso-occlusive crisis (VOC) which is alleviated through arginine supplementation in SCD patients [30].

L-arginine is converted to citrulline and nitric oxide (NO) by the enzyme NO synthase (NOS). Beneficial effect of NO has been observed in SCD patients having acute chest syndrome, hypoxia and pulmonary hypertension [31]. NO is pivotal in the expression of sickle cell VOC as it plays an important role in both vascular vasodilatation allowing blood to flow easily, and in reducing the adhesiveness of sickled erythrocytes to the vascular wall. It also reduces platelet aggregation and protects the blood vessels from free radicals [32]. Low level of L-arginine is observed in HbSS adults, which decreases further during severe pain episodes [33, 34]. Over expression of NO synthase under reduces arginine concentration, thereby producing ROS, increasing the oxidative stress and reducing NO availability in these patients [35]. Inhaled NO was found to reduce the time period and episode of an acute VOC in children affected with this disease. Sildenafil, which is an oral phosphodiesterase -5 inhibitor, is known to amplify the production of endogenous NO which in turn causes an increase in HbF production [36].

### Role of micronutrients

There is ample evidence linking lower levels of zinc with increased painful crisis in SCD patients. The increased daily zinc requirement in SCD patients, which is not met by the usual intake, is a consequence of hyper zincuria caused by increased hemolysis [37, 38].

Also alterations of several parameters of cellular immune functions in SCD patients have been related to a deficiency of zinc [37]. Zinc supplementation to pre pubertal aged sickle cell anemia patients was found to be beneficial on their linear growth and weight [39]. Zinc sulphate has been found quite effective in reducing RBC’s dehydration. It was also found to reduce sickle cell crises, pain and other life-threatening complications. Zinc supplementation not only improved growth and weight of SCD children, but also gave a boost to their immunity by offering antibacterial protection, thereby making its supplementation essential. Besides zinc, magnesium was found to protect against losses of water and potassium in SCD patients [39, 40]. Magnesium deficiency was found to increase episodes of sickling by causing cell dehydration in sickled erythrocytes [40]. In SCD patients It has been found that magnesium supplementation reduced the number of dense erythrocytes and also improved the erythrocyte membrane transport abnormalities of patients with SCD [41].

Copper deficiency is linked to anemia, being a key ingredient for functioning of metalloenzymes (e.g. ceruloplasmin), and plays an important role in iron metabolism. Ceruloplasmin helps to mobilize stored iron in the liver and make it more available for synthesis of Hb. Importantly, in copper deficiency anemia the synthesis rate of Hb is reduced, despite increased iron levels in the liver [42, 43].

Chromium is another element which helps in the management of SCD. It acts not only as a cofactor, at receptor sites of the insulin sensitive cell membrane but it also plays a role in carbohydrate metabolism which is the body’s much required energy source in SCD.

Manganese (Mn) which helps in glycoprotein synthesis and bone formation is also important for management of SCD. It also acts as cofactor of pyruvate carboxylase which participates in the respiratory chain reaction required for the much needed energy production, for SCD patients [44, 45].

### Role of vitamins

As compared to their peers, SCD children are known to show decreased height and weight resulting in poor growth. Blood deficiency of several vitamins such as A, B6, C, D,& E and minerals like zinc, Mg is observed in patients with SCD by many researchers, which are the most factors contributing towards their poor growth and weight [46, 47]. These deficiencies cause a significant reduction in the concentrations of blood antioxidant levels in SCD patients resulting in VOC related ACS [48]. Researches supplementing vitamins E and C and minerals, magnesium and zinc either alone or in combination proved beneficial outcome in reducing the sickling pain crisis [49]. The decrease in concentration of antioxidant vitamins A, C, and E is responsible for increased hemolysis and susceptibility to bacterial infections in SCD. Decreases in arterial blood pressure reduction in the percentage of sickle cells, was observed with a collective supplementation of vitamins like A, C, and E, besides an increase in concentration of hemoglobin and packed cell volume [50, 51]. In vitro studies also established that vitamin E possesses anti-sickling property and therapeutic benefits in sickle cell anemia cases [52]. As nutritional deficiency plays a significant role in SCD complication, educating the patients on specific nutrient and calorie needs should be emphasized. Proper care must be provided about their dietary intake, ways of providing nutritious meals (particularly among the low income group), and means for enhancing protein and calorie consumption [46].

Administration of 1 mg vitamin B12 intramuscularly for a period of around 3 months showed significant symptomatic improvement in SCD patients. This study also confirmed that in cases of severity, these patients may undergo unrecognized deficiency of vitamin B12 [53]. Most of the pediatric patients display an adequate status of vitamins B6 and B12, but raised levels of plasma homocysteine along with a low folate levels. Folate supplementation would be beneficial to these patients, as it lessens their risk for endothelial damage [20]. Vitamin B6 is known for its crucial roles in different metabolic pathways in the body like ingestionof food, derivatization to the fueling molecule glucose and assisting in the metabolism of lipids and proteins. The biological potent form of vitamin B6, namely pyridoxal 5’ phosphate (PLP) functions as a cofactor for many of the enzymes involved in amino acid metabolism as well as the formation of neurotransmitters like serotonin, dopamine, epinephrine, and gamma amino-butyric acid.

SCD patients are prone to bone fractures and often the vitamin D status is found to be very low, putting them at great risk. Routine supplementation with vitamin D helps maintain the vitamin levels in such patients [54].

### Proposed mechanisms for developing nutrient deficiencies in SCD

Proposed mechanisms of under nutrition may include dysfunctions in protein metabolism, cytokine related appetite suppression, enhanced cardiac energy output, and increases in erythrocyte levels [55, 56].

Hyper metabolism characterized by increased catabolism and lowered anabolism increases the calorific demand in SCD patients [57]. This is represented as increased resting energy expenditure (REE). A shortened lifespan of sickle cell blood cells causes an increased energy demand of the body. Hence their requirement of energy to sustain the normal functions of growth, physiological functioning and physical activity are not met. Increased energy requirements that are not followed by a concomitant calorific intake render an impaired growth status in these patients. Some researchers propose that frequent bouts of illnesses and hospitalization might have adverse effects on the frequency of food intakes and energy [55]. Also erythropoiesis increases the resting energy required in SCD. Researchers suggest that enhanced production of erythrocytes, increased cardiac output, and increased turnover of protein is possibly responsible for increased REE and protein requirements in SCD [58].

As SCD patients have lowered RBC concentration and are anemic, an elevation in resting cardiac indices is observed. In order to maintain and supply oxygen to the different tissues of the body, workload on the cardiac system is increased, thereby triggering a state of chronic inflammation. A study estimated the average hemoglobin synthesis to be ~ 0.725 g/kg/d in HbSS adults as compared to just 0.094 g/kg/d in their healthy counterparts. This results in an enhanced cell turnover in the bone marrow, and an increased glutamine uptake, thus leading to a depletion of the body’s glutamine levels. Further the study showed that pre-pubertal SCD children utilize 19 % more calories, about 58 % of more protein, and 47 % greater amount of glutamine than healthy children of their ages [59].

Large energy deficits due to a higher basal metabolic rate are regarded as one of the possible factors contributing towards poor growth in SCD individuals. This relatively low energy intake in younger children is also contributory to growth abnormalities [60]. Associations of protein intake and other macronutrients with growth measurements are limited due to studies that are not well structured and also smaller sample sizes. Protein utilization too appears compromised because of certain amino acid deficiencies. Hence the presence of orotic acid is observed in normal subjects with low intakes of protein which is similar to that observed in SCD patients [60].

Dysfunctional signaling of the hypothalamus and the inflammatory cytokines induced reduction in neuropeptide Y (NYP) release; appear to be responsible in causing changes in energy provided to the body. Inflammation may promote energy expenditure in a regulatory feedback manner to fight against energy supply in the peripheral organs/tissues as well as in the central nervous system. Blockage of the N YP leading to the suppression of appetite has been associated with noticeable weight loss and lowered physical activity. Other factors contributing to a reduced food intake may be also due to pain, fear, and other psychological stressors [61]).

### Role of interleukins

Higher levels of IL-1 and IL-6 have been linked with increased morbidity risk in SCD [62]. Patients with enhanced IL-6, C-reactive protein, IL-1β, and TNF-α exhibit threshold changes in their sense of taste,and odor thereby affecting their dietary intakes [63]. Animal experiments associate IL-1 concentrations with food intakes and satiety by triggering the neurons in the hypothalamus. Hence antagonist IL-1beta receptors have been found to be alleviated in anorexia [64].

Leptin is another pro inflammatory cytokine known to regulate the feeding behavior, and energy utilization [65]. The cytokine IL-6 known to modulate the levels of leptin has also been implicated in anorexia though another study shows contrary results. Weight loss has been shown to result in a lowered production of leptin which is proportional to the loss of body fat [66].

### Dysfunctional eating patterns

Psychological and social stresses, not to mention the extreme pain and the restricted activity, drive the SCD child to dysfunctional eating. Difficulty in eating and eating things not considered as food which are characteristic symptoms of pica, lead to nutritional deficiencies which go unnoticed by the clinician. Although one fourth of SCD children are affected by pica, it has not gained the focus of health care providers. Searching for biological explanations makes the clinician focus on aspects unrelated to psychological stressors [67].

### Dehydration

One of the important factors affecting cell sickling is the loss of water in the cell. The migration of potassium and chloride ions carrying water across the RBCs causes dehydration, which consequently increases the tendency of the hemoglobin to polymerize and sickle. Studies show a potential therapeutic solution to reduce the cell sickling by blocking the loss of electrolytes from erythrocytes through two ion transport pathways, namely the K-Cl co -transporter and the Ca2 + -activated K+ channel [68].

### Possible role of lipids

The role of dietary lipids deserves specific mention because, phospholipids composition of the membrane lipids in sickle cell erythrocytes show distinct abnormalities which may have a direct bearing upon the dehydration and abnormal sodium and potassium metabolism known to occur in sickle erythrocytes. This also has a direct impact on cellular function [69]. The increased permeability of sodium and potassium in the deoxygenated sickle cell leads to an overload on the renal system causing its damage [70]. In combination with the protein abnormality of hemoglobin, these molecular changes in membrane phospholipids composition may accentuate the sickling phenomena and perhaps, impart to the crisis in these patients.

### Potential nutritional approaches for SCD

Benefits from nutritional supplements in the management of SCD and its associated clinical conditions have strengthened considerations of nutritional aspects apart from the genetic one. Possible approaches for nutritional intervention to attain optimal immune and nutritional status for prevention of the related disease symptoms and reduction of morbidity and mortality in SCD patients are being explored. Use of dietary supplements to patients irrespective of age has shown improvements in growth and protection against infections [71].

### Traditional herbal therapy

Many herbs that contain beneficial phyto compounds have been used since ancient times to reduce sickling as well as the painful episodes. Leaf extracts of Carica papaya and Parquetina nigrescens, root extracts of Fagara zanthoxyloide*s*, and seed extracts of Cajanuscajan, contain phyto-antioxidants, which may act either alone or synergistically to augment the anti-sickling activity of these plants.(67) The Congolese plant Justicia secunda containing anthocyanins was found to exhibit potential antisickling effects [72, 73]. Eugenia caryophyllata and Piper guineense contain vanilloids like Shikimic acid and cannaboids that are of benefit in reducing the painful episodes by mechanisms similar to that of the opiates [74], F.zanthoxyloides, natively called fagara has been used for the reduction of painful episodes in SCD. Nicosan is a popular and safe Nigerian anti sickling herbal remedy inhibiting hemoglobin S polymerization [68]. Bioassay experiments show that maximum reduction in sickling was observed by the leaf extracts of C. papaya at concentrations of 5 mg/mL [52] probably due to relatively high total phenol content exhibiting high free radical scavenging activity. Though many of these phyto-medicines have proved beneficial, further multicentric researches are required to validate their usage.

### Antioxidant therapy

SCD is one of the many diseases in which oxidative stress plays a significant role affecting the RBCs, and leading to inflammation and the resulting pain. Levels of antioxidants seem to be compromised in SCD [75]. During management of clinical cases, the exacerbated iron load due to hemolysis and multiple transfusions potentiates ROS generation. Among several antioxidants known, the phenolic compounds isolated from plants, namely the flavonoids are very important. Supplementation of natural antioxidants vitamins such as A, C, and E have been shown to decrease the number of irreversibly sickled cells, the arterial blood pressure, and concentration of mean corpuscular Hb concentration with concomitant increases in packed cell volume (PCV} concentration [52].

The cyclic cascade leading to complications of cell adhesion and VOC, triggers production of ROS further intensifying the disease symptoms. As oxidative stress plays a pivotal role in SCD patients during VOC, the use of antioxidants to improve the clinical status seems essential. In-vivo studies on antioxidants using animal models have shown promising results. Ascorbic acid has shown effectivity as a potent antioxidant at as low as 0.1 millimolar concentrations [76]. A 10 week supplementation of vitamin E showed a decrease in the cell sickling from 25 % to 11 % [77].

However, clinical studies using vitamin E have shown poor success rates and found no significant differences in cell adhesion and lipid peroxidation levels [78]. This has prompted Ohnishi etal to advocate a mixture of antioxidants in the suppression of SCD symptoms [20]. It has been suggested that the generation of free radicals in SCD may overpower the strength of exogenous antioxidants and therefore a mixture of antioxidants may prove beneficial. Also the generation of ROS due to external factors of diet, environment, and co-morbidities associated with SCD, needs to be well explored and evaluated. Well defined studies showing effects of the two different types of antioxidants on severity of symptoms in SCD should be performed. Antioxidants providing enzyme defense (such as SOD, catalase, GPX, and hemo- oxygenase -1) and antioxidants scavenging different free radicals (such as vitamins E,C, GSH) should be independently considered [79]. Also in spite of enhanced levels of antioxidant intake in SCD is being advocated so far, no established standardization exists. Therefore it becomes difficult to generalize the results of a particular study for antioxidant usage or future therapy [80]. Future research should target specific ROS generation depots using different antioxidant combinations to reduce SCD complications. However, limited phase three clinical trials are available due to the complex pathophysiology in SCD [77].

### Use of dietary lipids and omega- 3 fatty acids

The popular lipid food additive butyric acid has been found to increase levels of HbF in the blood and stabilize the mental mood. Experimental evidence suggests that the two short chain fatty acid derivatives namely α methyl hydrocinnamic acid and 2, 2 dimethyl butyrate offer significant therapeutic benefits as they induce fetal γ globin expression in SCD. They are further found to stimulate HbF cells with erythroid proliferation at quite low doses than the existing phenyl butyrate and butyrate [81].

A striking enhancement in the concentration of lipid constituents in sickle cells is observed when matched with similarly aged erythrocytes. Also the erythrocytes left unsickled, occupy a larger surface area, appearing flattened as compared to normally aged cells [82]. Abnormalities in the membrane lipid composition in SCD appear to have a direct impact on the cellular functions. Phospholipid composition of the membrane in sickle cell erythrocytes shows distinct abnormalities [46]. Deficiencies of certain polyunsaturated fatty acids and their subsequent replacement by monounsaturated and saturated fatty acids may be related to dehydration and the uncharacteristic sodium and potassium transport in sickled erythrocytes. This could not only help in possible corrections through exogenous supplementation of these fatty acids, but also serve as a useful diagnostic marker [46]. Pilot studies suggested that supplements containing omega-3 fatty acids may decrease the painful hemolytic and VOC and improve the membrane fatty acid composition [18]. The omega-3 fatty acids namely eicosapentaenoic acid (EPA), and docosahexaenoic acid(DHA), are two important structural and functional constituents of the RBCs which have been shown to inhibit haemolysis and vasoocclusion, thereby reducing the number of VOC in SCD [18, 83, 84]. In addition, omega-3 fatty acids diminish the expression of intercellular adhesion molecule-1, leukocyte adhesion to vascular endothelium, and the production of four biologically active molecules involved in the pathophysiology of tissue damage in SCD,namely the interleukins (IL)-1*β*, IL-6, and IL- 8 and tumor necrosis factor-alpha [85]. Furthermore, it has been observed that the greater the quantity of EPA and DHA in the blood, the lower the risk of developing complications of SCD and the lesser the degree of anemia [86, 87]. Their affordability over other current therapies in non-affluent countries where the overwhelming majority of people affected by sickle cell disease live makes it a feasible option. The diverse anti-aggregatory, anti-adhesive, and anti-inflammatory role of omega-3 fatty acids makes it a promising therapeutic candidate for the prevention of cell sickling and reduction of the painful crisis in SCD [18].

### Use of amino-acids

Antisickling action of many plants of medicinal value and other naturally existing compounds may be attributed to the presence of amino acids [88]. Phenylalanine present in an herbal plant Cajanuscajan, and hydroxybenzoic acid present in another medicinal plant of the Vitex family are thought to be the reason for their antisickling effect [89]. Amino acids display their antisickling behavior by increasing the cell volume of the RBC’s, thereby decreasing the concentration of intracellular hemoglobin below the gelling threshold [90]. The possible mechanism of action leading to the antisickling effect by phenylalanine, is reported to involve the liposomal transport system, as well as the Na+/K+ transport system [91]. The aromatic compound L-phenylalanine benzyl ester acts as an antisickling agent, and is found to possess potential therapeutic properties beneficial in the treatment of SCD [92].

### Use of opioids

Individuals addicted with opiates often experience severe macro and micro nutrient deficiencies which renders their protein and carbohydrate metabolism inefficient. Being indifferent to the basic necessities of life makes them impoverished and undernourished. This makes them underweight with a lowered immunity, hormonal imbalances, prone to infections and organ damage. Alterations in the levels of certain specific nutrients may also hinder their addiction withdrawal. Moreover lack of nutritional education leads them to unhealthy eating behaviors. Unfortunately addiction centers do not identify nutritional programs as a major influencing factor. Effective supplementation of large amounts of protein and amino acids during de addiction, may be required to boost their nutritional status [93].

Opoids exhibit their beneficial anti analgesic activity by either exerting their histaminergic effect, excitatory effect, dopaminergic effect or proserotonergic effect [94]. A fear of addiction to narcotics by patients, giving rise to ethical issues [95] has also led to its controlled usage. Use of intranasal fentanyl in a pediatric emergency setting was related with a considerably reduced time to attain analgesia as compared with morphine, administered parenterally, and caused minimum distress to children [96] Experiences from managing pain in children could well be then extrapolated to adults.

### Current new approaches

In response to anemia, the kidneys secrete erythropoietin which is the major non-immunological cytokine that regulates erythropoiesis. Therefore serum levels of erythropoietin are often increased in SCD wherein chronic anemia is observed [94]. Erythropoietin’s ability to provide a stimulus in HbF production could be used for clinical trials. This raise in HbF gives us a lead that human erythropoietin could be used favorably in SCD as it either increases availability of oxygen and nutrients or produces proteins favoring growth and healing. This is also observed in supplementation with HU [53].

The anti-fungal compound clotrimazole, appears to block the Ca2 + -activated K+ channels in RBCs and in smooth muscle cells via the cytochrome P450 [97]. Researches are on to experiment this compound in conjunction with other bioactive compounds for long term treatment.

Ozone being the allotrope of oxygen, possesses healing properties which are yet to be completely understood. Bocci et al. observed that ozone activates a number of biochemical pathways, which are very useful in vasculopathies, particularly chronic limb ischemia [98]. The rationale for using ozone therapy in SCD appears to be based upon its action on the endothelium thereby enhancing the release of NO and prostacyclin and suppressing the release of endothelin-1(a vaso constriction affecting peptide that is increased during cell injury and insult) [99]. Careful blood ozonization appears to be one of the few effective procedures for correcting chronic oxidative stress [99].

Significant progress has been achieved in gene therapy approaches for treatment of SCD [100]. Gene therapy in SCD patients has been known to target major repressor proteins implicated during development [10]. Gene therapy approaches in SCD mice decreased anemia and hyper leukocytosis, reduced the accumulation of iron in liver, and enhanced splenic and renal function. Thus, modest chimerism with the donor cells exhibiting high levels of HbF from a γ-globin lentiviral vector that is insulated can mend the pathological status in SCD mice. This would render a safer and effective option of gene therapeutics in humans [101]. Talano et al. have shown the usage of alternative allogeneic donors which can either be familial haploidentical (FHI) donors, matched unrelated donors (MUD), or unrelated cord blood donors (UCB). These therapies have ensued in high survival rate among SCD patients. Now due to these advanced approaches, a lot of people having chronic SCD conditions are able to receive curative allogenic stem cell therapies [16].

Newer non-invasive therapies like lung extension to ease the airway pressure, pulse oximetry, and incentive spirometry hold potential for future treatments [102]. Continuous positive airway pressure (CPAP) therapy treated subjects exhibited lesser pain, improved cognition, and a decreased apnea-hypopnea index. The intensity of pain days was reduced from 2.3 days to 0.8 days per week for each patient [103]. A dysfunction in the hemoglobin of SCD and a right-shifted oxyhemoglobin dissociation curve (ODC) alter the oxyhemoglobin saturation (SpO_2_) values that can be measured by pulse oximetry which is a non-invasive method [104]. Incentive spirometry [105],as well as positive expiratory pressure therapies also showed a great promise especially in children who may experience hypoventilation when administered high doses of opiates for painful VOC crisis [106].

## Conclusion

SCD is well known to be an inherited disorder with a disruptive metabolic status causing immense pain and a compromised life for the patient. Different patterns of culture, unmet economic needs, lack of health education and nutritional awareness place the sickle cell disease patient to increased risk of dietary deficiencies leading to pain and problems. These nutritional deficiencies may surface either due to lowered intake of specific nutrients or malfunctioning in the metabolic pathway or alterations at the genetic level.

Though HU therapy is clinically very promising, certain areas like lowering of sperm counts in males [107], instability in the genetic makeup through deregulation of telomere repeat binding factor 2 (TRF2) and telomere dysfunction [108] is a matter of concern. This calls for further researches for compounds to alleviate the pain and improve the condition of patients with SCD. Nutrients with their immense benefits could add to the prevention and curative aspects of SCD. Also translation of genetic technology,particularly epigenetic nutrition could well pave the way to better SCD patient care [109].

Though much has been reported about nutritional supplements and their benefits, little has changed in clinical practice. Also larger well-structured multi-centric studies need to be addressed to arrive at conclusive evidence-based recommendations by a scientific body. This would then gain the much required attention on nutritional aspects by clinicians, and benefit the SCD patient.

## Abbreviations

ACS: Acute chest syndrome
DHA: Docosahexaenoic acid
EPA: Eicosapentaenoic acid
HbF: Fetal hemoglobin
HSCT: Hematopoietic stem cell transplantation
HU: Hydroxyurea
IL: Interleukins
NO: Nitric Oxide
REE: Resting energy expenditure
ROS: Reactive oxygen species
SCD: Sickle cell disease
VOC: Vaso occlusive crisis
